# The Effects of Anthocyanins and Their Microbial Metabolites on the Expression and Enzyme Activities of Paraoxonase 1, an Important Marker of HDL Function

**DOI:** 10.3390/nu11122872

**Published:** 2019-11-24

**Authors:** Hassan T. Aboufarrag, Paul W. Needs, Gerald Rimbach, Paul A. Kroon

**Affiliations:** 1 Food Innovation & Health, Quadram Institute Bioscience, Norwich Research Park, Norwich, Norfolk NR4 7UA, UK; hassan.aboufarrag@quadram.ac.uk (H.T.A.); paul.needs@quadram.ac.uk (P.W.N.); 2 Food Science and Technology Department, Faculty of Agriculture, Alexandria University, Alexandria 23511, Egypt; 3 Institute of Human Nutrition and Food Science, Christian-Albrechts-University of Kiel, 24118 Kiel, Germany; rimbach@foodsci.uni-kiel.de

**Keywords:** polyphenols, flavonoids, phase-II metabolism, anthocyanin metabolites, human metabolism, lactonase activity, arylesterase activity, promoter activity, single nucleotide polymorphism, protocatechuic acid, phloroglucinaldehyde

## Abstract

High circulating HDL concentrations and measures of various HDL functions are inversely associated with cardiovascular disease (CVD) risk. Paraoxonase 1 (PON1) contributes to many of the athero-protective functions of HDL, such as promoting the reverse cholesterol transport process and reducing the levels of oxidized LDL. PON1 activities are influenced by several factors, the most important being diet and genetic polymorphisms. Reported data from randomized controlled trials have shown that anthocyanin consumption increased PON1 activity. However, the underlying molecular mechanisms by which anthocyanins increase PON1 activity are not understood. Therefore, the aim of this research was to investigate the ability of anthocyanins and their metabolites to increase *PON1* gene expression and/or enzyme activities as potential mechanisms. The effect of the two predominant dietary anthocyanins and 18 of their recently identified microbial metabolites including their phase-II conjugates on *PON1* gene expression was studied using a PON1-Huh7 stably-transfected cell line and reporter gene assay. The effects of these compounds on PON1 arylesterase and lactonase activities were investigated using two isoforms of the PON1 enzyme that are the phenotypes of the 192Q/R polymorphism. None of the compounds caused even modest changes in PON1 promoter activity (*p* ≥ 0.05). Further, none of the compounds at physiological concentrations caused any significant changes in the arylesterase or lactonase activity of either of the iso-enzymes. Cyanidin reduced the lactonase activity of the PON1-R192R enzyme at high concentrations (−22%, *p* < 0.001), but not at physiologically achievable concentrations. In conclusion, none of the data reported here support the notion that anthocyanins or their metabolites affect PON1 transactivation or enzyme activities.

## 1. Introduction

Many epidemiological and clinical studies suggest that the consumption of anthocyanin-rich fruit and vegetables is associated with favorable improvements in lipid profiles, specifically with reductions in LDL cholesterol and increases in HDL cholesterol [1,2,3,4]. High levels of circulating HDL cholesterol are inversely correlated with the incidence of cardiovascular disease (CVD), mainly coronary heart disease (CHD) [5,6,7]. However, more recent studies have suggested that the relationship between HDL and CVD markers extends beyond the concentration of HDL alone and that the function of HDL may be more important than HDL concentration in protecting against CVD [8,9,10,11]. HDL possesses a number of atheroprotective functions such as mediation of cholesterol efflux from cholesterol-loaded cells, protection against oxidation and inflammation, and promotion of nitric oxide synthesis [12]. This protective effect is largely attributed to enzymes associated with HDL, including paraoxonase 1 (PON1) [13].

PON1 is an HDL-associated enzyme secreted by the liver and found to have significant anti-oxidative and anti-inflammatory properties though its lactonase, peroxidase, and esterase activities [14]. The anti-atherogenicity of PON1 is thought to be related to its ability to hydrolyze oxidized cholesteryl esters and oxidized phospholipids and degrade hydrogen peroxide, thus protecting lipoprotein particles from any further oxidative modification [15].

Polymorphisms in the *PON1* gene can affect enzyme activities, stability, and the anti-atherogenicity of the PON1 enzyme [16,17,18,19,20]. Among the numerous PON1 polymorphisms in humans, the Q192R and L55M polymorphisms are the ones most associated with lipoprotein oxidation and CHD risk, and there is evidence that these polymorphisms explain a significant proportion of the differences in PON1 activity between individuals [21]. People with the 192-Q/Q genotype gain greater protection against CVD compared to those with 192-R/R PON1. The 192-Q/Q PON1 enzyme is more potent in decreasing the levels of oxidized lipids in human atherosclerotic lesions than the 192-R/R PON1 enzyme [22,23]. The PON1 L55M polymorphism has also been associated with variation in serum PON1 activity but has a weaker effect [24]. PON1 polymorphisms also affect the enzyme’s substrate specificity [25]. For instance, the 192-R/R PON1 enzyme hydrolyses paraoxon approximately nine times faster than the 192-Q/Q, PON1 enzyme, while the opposite occurs with diazoxon and sarin substrates [25]. Therefore, ignoring the genetic variant could lead to a false interpretation, especially, when substrates that are strongly influenced by polymorphisms such as paraoxon are being used [26]. Therefore, it is recommended to compare PON1 levels within each different genotype/phenotype group.

Anthocyanins have been reported to increase PON1 activity. A 17.4% mean increase in PON1 arylesterase was reported in response to a 24-week intervention with a mixture of purified anthocyanins extracted from bilberry and blackcurrant (Medox™) in human participants with hypercholesterolemia compared to placebo [27]. A similar effect on serum PON1 was reported for participants who had consumed pomegranate juice for two weeks compared to a control beverage [28]. In addition, treatment of PON1-Huh7 cells with polyphenol-rich and anthocyanin-rich purple sweet potato fractions was reported to cause significant induction of PON1 promoter transactivation [29]. Other polyphenols such as quercetin, resveratrol, and catechin have also been reported to modulate PON1 activity and gene expression in vivo and in vitro [30,31,32,33,34,35].

There is growing evidence that anthocyanins are subjected to extensive metabolism, especially by the gut microbiota, producing a wide range of metabolites [36]. After consumption of penta-^13^C-labelled cyanidin-3-glucoside (C3G), most of the given dose was recovered as breakdown products (A- and B-ring-derived phenolics), while only minor quantities of intact C3G were recovered [37,38]. Of the 35 metabolites identified in human plasma, urine, and feces in this study, hippuric acid, vanillic acid, ferulic acid, 4-hydroxybenzaldehyde, and vanillic acid sulphate were the predominant metabolites [38]. The much higher concentrations in blood and the relative stability of anthocyanin metabolites suggest that the high bioactivity of anthocyanins is more likely to be mediated by their metabolites rather than the parent compounds. However, the biological activity of the majority of these metabolites has not been investigated.

Therefore, the aim of this study was to investigate the effects of two predominant dietary anthocyanins (C3G and delphindin-3-glucoside (D3G)), and their degradation products including their phase-II conjugates on *PON1* gene expression and PON1 arylesterase and lactonase activities. The potential for anthocyanins or their metabolites to induce PON1 expression was determined using a reporter gene assay and the effects on enzyme activities assessed using isolated PON1 isoenzymes corresponding to the major functional SNPs in humans.

## 2. Materials and Methods

### 2.1. Reporter Gene Assay

#### 2.1.1. Cell Culture

The effect of anthocyanins and their metabolites on *PON1* gene expression was evaluated using a reporter gene assay (Bioluminescence firefly luciferase assay) in cultured hepatocytes. PON1-Huh7 was a Huh7 liver hepatoma cell line that had been stably transfected with a reporter plasmid containing 1009 bp [–1013, –4] of the *PON1* gene promoter cloned into the firefly luciferase reporter vector pGL3 basic [30,39].

PON1-Huh7 cells were kindly provided by Dr. X. Coumoul and Dr. Robert Barouki, French National Institute of Health and Medical Research (INSERM), France. PON1-Huh7 cells were maintained following the protocol as previously described [39]. Briefly, PON-Huh7 cells were cultivated in Dulbecco’s Modified Eagle’s Medium (DMEM) supplemented with fetal bovine serum (FBS) 10% (v/v), penicillin (100 U/mL) and streptomycin (100 µg/mL), glutamine (2 mM), and G418 disulphate (100 µg/mL) (Sigma-Aldrich) at 37 °C in 5% CO_2_ until reaching 80%–90% confluency, with the media changed every two days. The cells were then detached by adding trypsin-EDTA for 2–3 min at 37 °C. A complete DMEM that contained 10% FBS was then added to inhibit the trypsin. The trypsinized cell suspension was collected and the cells were either split or seeded for treatments. In 24-well plates, cells were seeded at an initial density of 0.15 × 10^6^ cells/well and left to attach for 24 h.

#### 2.1.2. Treatments

**Figure 1:**
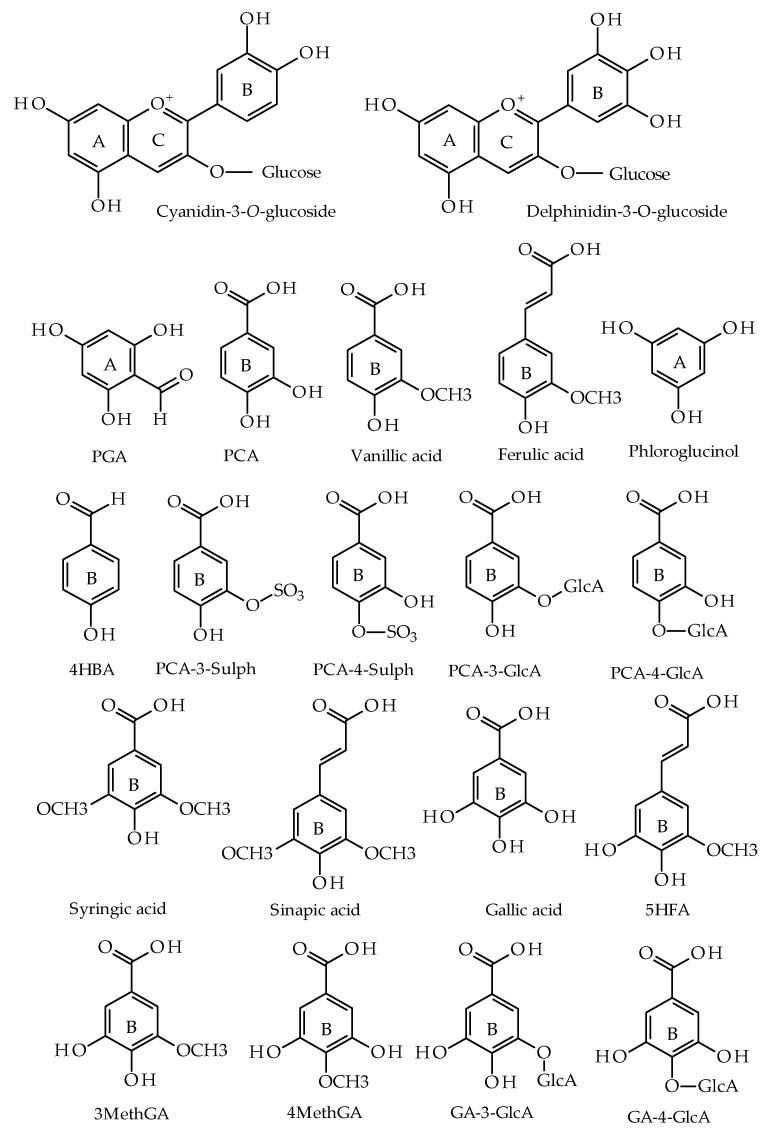
The chemical structures of cyanidin-3-*O*-glucoside, delphinidin-3-*O*-glucoside, and their known and potential metabolites: phloroglucinaldehyde (PGA), protocatechuic acid (PCA), 4-hydroxybenzaldehyde (4HBA), PCA-3-sulphate (PCA-3-sulph), PCA-4-sulphate (PCA-4-Sulph), PCA-3-glucuronide (PCA-4-GlcA), PCA-4-glucuronide (PCA-4-GlcA), 3-*O*-methylgallic acid (3MethGA), 4-*O*-methylgallic acid (4MethGA), gallic-3-glucuronide (GA-4-GlcA), and gallic acid-4-glucuronide (GA-4-GlcA). AccelrysDraw (version 4.2 for windows) was used to draw the chemical structures.

Following seeding the cells, the media was removed and new pre-warmed media containing anthocyanins treatment was added to the appropriate wells and incubated at 37 °C, 5% CO_2_ for 48 h. The treatments were C3G, D3G, and their major known human metabolites (reported elsewhere [38]) together with their potential/predicted metabolites at 1 and 10 µM. The cells were also treated with phase-II conjugates of protocatechuic acid (PCA) and gallic acid, namely PCA-glucuronides (PCA-GlcAs, including PCA-3-glucuronid (PCA-3-GlcA) and PCA-4-glucuronide (PCA-4-GlcA), each at 1 µM), PCA-sulphates (PCA-sulphs, including PCA-3-sulphate (PCA-3-Sulph) and PCA-4-sulphate (PCA-4-Sulph), each at 1 µM), gallic acid glucuronides (GA-GlcAs, including gallic acid-3-glucuronide (GA-3-GlcA) and gallic acid-4-glucuronide (GA-4-GlcA), each at 1 µM), and methylgallates (MethGA, including 3-O-methylgallic acid (3MethGA) and 4-O-methylgallic acid (4MethGA), each at 1 µM). Figure 1 depicts the chemical structure of these metabolites. In order to investigate the interaction between anthocyanin metabolites, the cells were treated with two different mixtures of anthocyanin metabolites, PCA-Mix and GA-Mix. PCA-Mix contains C3G, PCA, PCA-4-GlcA, PCA-3-GlcA, PCA-3-Sulph, and PCA-4-Sulph, at 1 µM each. GA-Mix contains D3G, gallic acid, GA-3-GlcA, GA-4-GlcA, 3MethGA, and 4MethGA, at 1 µM each. The vehicle control was DMSO with final concentration of 0.1%. Curcumin (20 µM) (Fisher Scientific) served as a positive control alongside the anthocyanins and their metabolite treatments. Treatments were conducted in quadruplicate and the experiments were repeated at least two times. The tested compounds were purchased from Sigma Aldrich, syringic acid was purchased from Alfa Aesar, and phloroglucinol was purchased from Across Organics. PCA conjugates and gallic acid conjugates were synthesized in-house [40]. After treatment, the luciferase activity was measured as a marker of PON1 promoter activity.

#### 2.1.3. Luciferase Assay

Luciferase activity was measured using the Luciferase Assay System (Promega, Hampshire, UK, Cat# E1500) according to the manufacturer’s instructions. Briefly, after 48 h treatment, the media was removed, and the cell layer was washed twice with cold calcium- and magnesium-free PBS. The lysis buffer was then added to the cell layer and the cells were scraped. Immediately, the lysate was collected and centrifuged for 2 min at 12,000 XG at 4 °C. After that, in 96-well white plates, 100 µL of luciferase assay reagent were added to 20 µL cell lysate. The luminescence was immediately measured over 20 s using a plate reader, FLUROstar Optima (BMG labtech, UK). 

The plate reader is capable of injecting the reagent automatically and can perform multi-well readings. The luminescence was expressed as total light intensity which was collected over 20 s. Results were normalized to the total cell protein content. The total protein in each sample was determined using the bicinchoninic acid (BCA)-Reducing Agent Compatible assay as per the manufacturer’s protocol (Thermofisher, Paisley, UK, Cat# 23252).

#### 2.1.4. PON1 Promoter Activity Calculation

First, the light intensity which reflects the promoter activity was normalized to the total protein content in the cell lysate.

Promoter activity = Total light intensity/total protein content (µg/mL).

Then, the fold change of PON1 promoter activity was calculated relative to the control (DMSO).

#### 2.1.5. PON1 Enzyme Activities

The direct effect of anthocyanins on PON1 activities was measured using a commercially available purified PON1 that phenotyped into two phenotypes based on Q192R polymorphism. PON1 phenotype QQ (PON-QQ) and phenotype RR (PON-RR) were purchased from ZeptoMetrix (Buffalo NY, USA, Cat# 0801384). The treatments were prepared as described earlier and were diluted in the assay buffer. Arylesterase and lactonase PON1 activities were measured using colourimetric assays as described below.

#### 2.1.6. PON1 Arylesterase

PON1 arylesterase activity was quantified by measuring the hydrolysis rate of p-nitrophenyl acetate (a colorless substrate) into p-nitrophenol (that has a yellow color) and measuring the increase in absorbance using a spectrophotometer [41]. PON1 arylesterase activity was measured as described elsewhere [13,42,43]. First, PON-RR and PON-QQ were diluted in assay buffer consisting of 20 mM Tris-HCl buffer, pH = 8 and 1 mM CaCl_2_, just prior to conducting the assay. The dilution factors for PON-RR and PON-QQ were 10- and 15-fold, respectively. The assay was developed with a final volume of 200 µL in 96-well polystyrene plate. Here, 20 µL of diluted enzyme and 20 µL of treatment were mixed together with 140 µL pre-warmed assay buffer. The plate was then sealed to prevent evaporation and incubated at 37 °C for 10 min. Quickly, 20 µL of diluted substrate was added to the previous reaction mixture and the increase in absorbance was measured immediately at 410 nm for 10 min using FLUROstar Optima (BMG labtech, UK). The final concentrations of reactants were 1% PON-RR (0.63 U/mL)/0.7% PON-QQ (1.3 U/mL), 1 mM p-nitrophenyl acetate, and 1 or 10 µM anthocyanin treatments. The final concentration of DMSO was 0.1% in all treatments. The control treatment was enzyme treated with DMSO at final concentration of 0.1%. The slope of the reaction rate was calculated using the instrument software. Blanks without enzymes or treatments were used to correct for the spontaneous nonenzymatic hydrolysis of p-nitrophenyl acetate by subtracting blanks from the hydrolysis rate of treatments. The % of change was calculated relative to the control as following:

$$\%\;of\;Change\;=\;\frac{hydrolysis\;rate\;of\;treatment-hydrolysis\;rate\;of\;control}{hydrolysis\;rate\;of\;control}\;\times \;100$$

Treatments were conducted in triplicates and the experiments were repeated at least two times. The purity of the enzymes was determined by measuring the activity of enzymes with 100 µM of 2-hydroxyquinoline (2-HQ), the PON1-potent inhibitor (Sigma-Aldrich, Dorset, UK, Cat# 270873).

#### 2.1.7. PON1 Lactonase

PON1 lactonase activity was measured spectrophotometrically by monitoring the hydrolysis of the synthetic lipolactone substrate, 5-thiobutyl butyrolactone (TBBL) [44,45]. Lactonase activity was measured as described previously [46,47]. Briefly, PON-RR and PON-QQ were diluted to 50- and 80-fold, respectively, in the assay buffer that consisted of 50 mM Tris-HCl, 1 mM CaCl_2,_ 50 mM NaCl, and pH = 8. Using 96-well polystyrene plates, 20 µL of enzyme and 20 µL of treatment were mixed together with 60 µL pre-warmed assay buffer, and then 50 µL of 5,5′-dithiobis (2-nitrobenzoic acid) (DTNB, 4 mM) were mixed with the reaction mixture. The plate was sealed to prevent evaporation and incubated for 10 min at 37 °C. A solution of TBBL (2 mM) was prepared in pre-warmed assay buffer contain 2% acetonitrile just before use. After that, 50 µL of TBBL (2 mM) was added to the reaction mixture and the absorbance was measured immediately at 412 nm using a FLUROstar Optima plate reader (BMG labtech, UK). The final volume was 200 µL and the concentrations of reactants were 0.2% PON-RR (0.13 U/mL)/0.125% PON-QQ (0.23 U/mL), 1 mM DTNB, 0.5 mM TBBL, and 1 or 10 µM treatments. The lactonase activity was calculated as previously described for arylesterase. Treatments were conducted in quadruplicate and the experiments were repeated at least two times. The purity of the enzymes was determined by measuring the activity of enzymes with 100 µM of 2-hydroxyquinoline (2-HQ).

#### 2.1.8. Statistical Analysis

All data and statistics were analyzed using GraphPad Prism (version 5.04 for Windows, GraphPad Software, La Jolla California USA, (https://www.graphpad.com/). All values are given as means ± SD. Any statistical difference between the groups was determined with one-way ANOVA coupled with Dunnett’s multiple comparison test comparing all sample groups to control (DMSO 0.1%). Values of *p* ≤ 0.05 were considered significant.

## 3. Results

### 3.1. Effects of Anthocyanins and Their Metabolites on PON1 Promoter Activity

**Figure 2:**
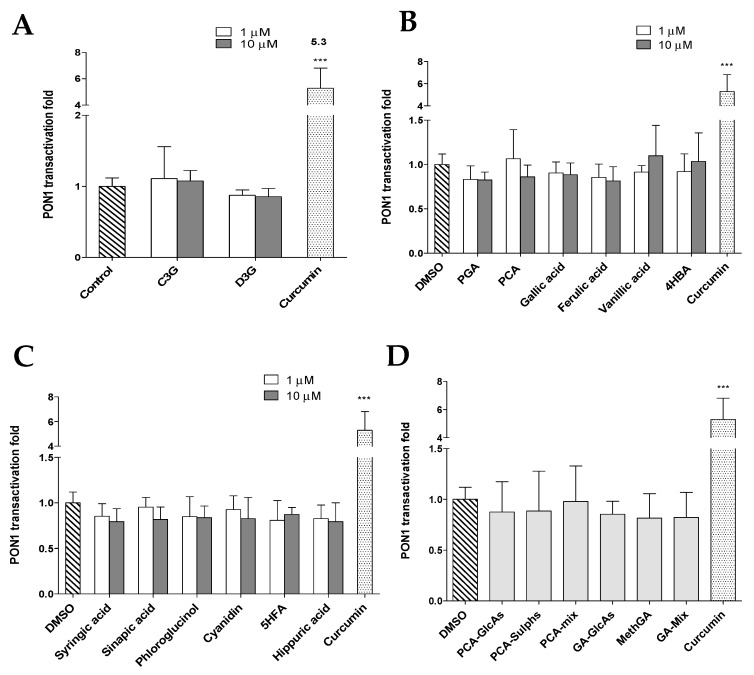
Effect of anthocyanin parent compounds (**A**), their known human metabolites (**B**), their potential/predicted metabolites (**C**), and phase-II conjugates (**D**) on paraoxonase 1 (PON1) promoter activity. A reporter gene assay was used to measure the promoter activity in PON1-Huh7 cells. The fold change was calculated relative to control (DMSO). Data are shown as means ± SD. *** *p* ≤ 0.001 as compared to control using one-way ANOVA coupled with Dunnett’s multiple comparison test. Treatments were as follows: cyanidin-3-glucoside (C3G), delphinidin-3-glucoside (D3G), phloroglucinaldehyde (PGA), protocatechuic acid (PCA), 4-hydroxybenzaldehyde (4HBA), 5-hydroxyferulic acid (5HFA), PCA-glucuronides (PCA-GlcAs), PCA-sulphates (PCA-Sulphs) PCA-Mix (C3G, PCA, and PCA conjugates), gallic acid glucuronides (GA-GlcAs), methylgallates (MethGA), GA-Mix (D3G, gallic acid and gallic acid conjugates), and curcumin (20 µM) which served as a positive control. Treatments were conducted in quadruplicate and the experiments were repeated at least two times.

The effect of anthocyanins and their metabolites on *PON1* gene expression was investigated by measuring activation of the PON1 promoter in PON1-Huh7 stably transfected cells. As shown in Figure 2A, C3G and D3G did not cause any significant change in PON1 promoter activity compared to the control (DMSO) at either 1 or 10 µM; C3G treatment resulted in a small increase but this was not significant (*p* ≥ 0.05). Similarly, a large number of anthocyanin metabolites were tested, including known metabolites of anthocyanins that were reported previously from the study that used penta-^13^C-labelled C3G [38] (Figure 2B) and a series of predicted anthocyanin metabolites. None of these metabolites (Figure 2C) or the phase-II-conjugates (Figure 2D) that were tested had any significant effect on PON1 promoter activity. In contrast to what we had predicted, syringic acid, cyanidin, 5-HFA, and hippuric acid reduced promoter activity by between 10% and 20%, although the changes were not significant (Figure 2C). In contrast, curcumin (the positive control) significantly increased PON1 promoter activity by 5.3-fold (Figure 2A–D, *p* ≤ 0.001) which was consistent with previous reports [29,39,48]. This demonstrates that the model is functioning properly and sensitive to treatments. Although these data showed that anthocyanins and their metabolites did not significantly alter the promoter activity of PON1, it is possible that they may interact with PON1 in a different way, for example by activating the enzyme and increasing its activities. Therefore, the effect of anthocyanins and their metabolites on PON1 enzyme activities were investigated.

### 3.2. Effect of Anthocyanins and Their Metabolites on PON1 Activities

#### 3.2.1. Establishing a Fit-for-Purpose Enzyme Assay

**Figure 3:**
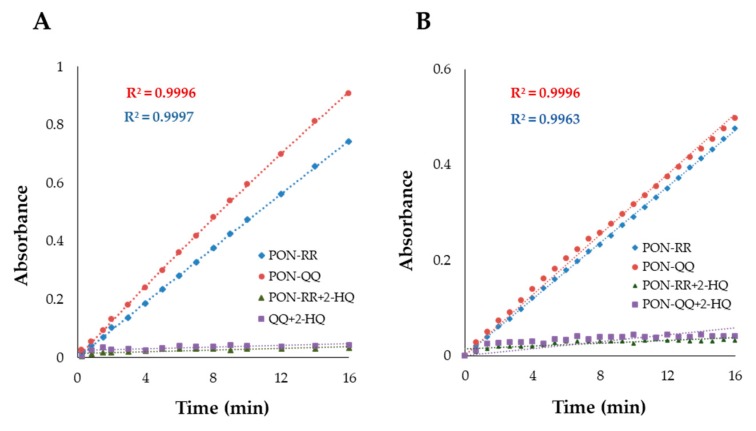
PON1 enzyme reaction progress curve for arylesterase (**A**) and lactonase (**B**). For arylesterase, the substrate was added to the 1% PON1 R192R phenotype (PON-RR) and to the 0.7% PON1 Q192Q phenotype (PON-QQ) with and without PON1 inhibitor (100 µM 2-hydroxyquinoline (2-HQ)). For lactonase, the substrate was added to 0.2% PON-RR and 0.125% PON-QQ with and without 100 µM 2-HQ. The enzymes were diluted in assay buffer. The absorbance was recorded over 16 min. R^2^ = linear regression coefficient. R^2^ was calculated using Excel software 2016.

To avoid inaccurate enzyme activity measurements, the reaction rate should be measured within the linear stage of the enzyme reaction. To establish a linear enzyme reaction, serial dilutions of PON-RR and PON-QQ enzymes were tested with p-nitrophenyl acetate (the substrate for arylesterase) and TBBL (the substrate for lactonase). A concentration of 1% PON-RR (0.63 U/mL) and 0.7% PON-QQ (1.3 U/mL) enzymes resulted in a linear reaction of arylesterase (Figure 3A), while the linearity of the lactonase enzyme reaction was achieved with 0.2% PON-RR (0.13 U/mL) and with 0.125% PON-QQ (0.23 U/mL) (Figure 3B). The linear regression coefficient (R^2^) was about 0.99, which is very close to 1 for both arylesterase and lactonase, meaning that the reaction is linear at these concentrations of PON-RR and PON-QQ. In addition, the reaction remained linear for at least 16 min for both reactions (Figure 3A,B). To make sure that the model was working and to validate that the commercial enzyme does not contain any other esterases rather than PON1, the arylesterase and lactonase activities were measured in the presence and absence of 2-hydroxyquinoline (2-HQ), a potent inhibitor of PON1. It was found that 2-HQ resulted in about 97.5% inhibition for PON-RR and PON-QQ for both activities, confirming the suitability of the model for this purpose of study (Figure 3A,B).

#### 3.2.2. Effect of Anthocyanins and Their Metabolites on PON1 Arylesterase Activity

**Figure 4:**
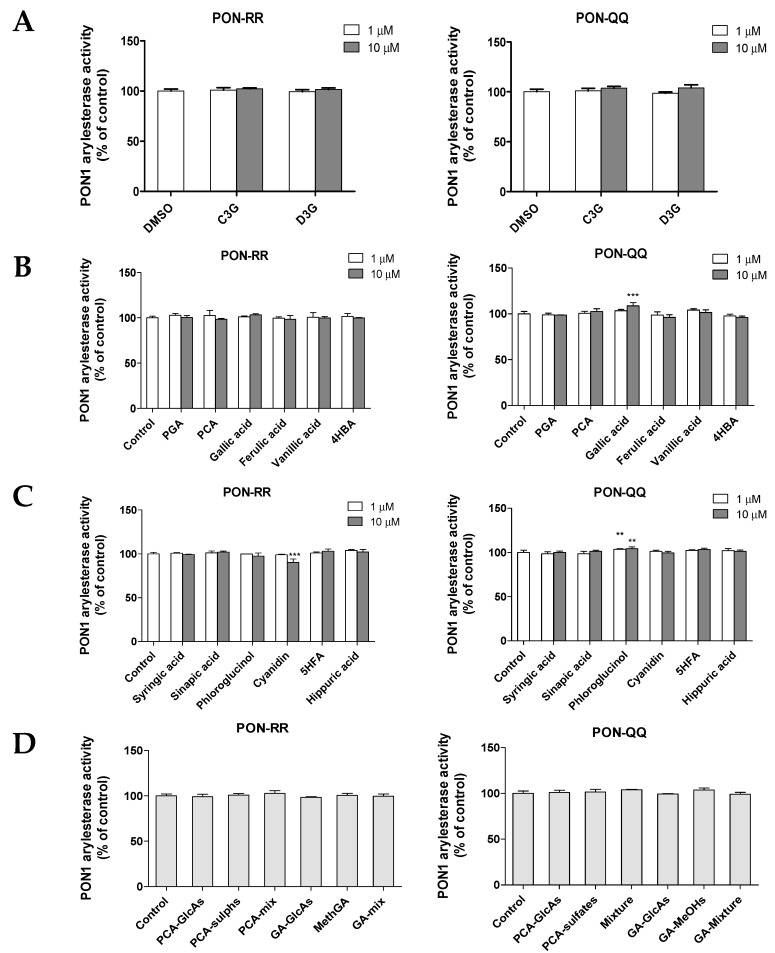
Effect of anthocyanins parent compounds (**A**), their known human metabolites (**B**), their potential/predicted metabolites (**C**), and phase-II-conjugates (**D**) on arylesterase activity of PON1 R192R (PON-RR) and Q192Q (PON-QQ) phenotypes. The % of change in activity was calculated relative to the control (DMSO). Data are shown as means ± SD. ** *p* ≤ 0.01 and *** *p* ≤ 0.001 as compared to control using one-way ANOVA coupled with Dunnett’s multiple comparison test. Treatments were: cyanidin-3-glucoside (C3G), delphinidin-3-glucoside (D3G), phloroglucinaldehyde (PGA), protocatechuic acid (PCA), 4-hydroxybenzaldehyde (4HBA), 5-hydroxyferulic acid (5HFA), PCA-glucuronides (PCA-GlcAs), PCA-sulphates (PCA-Sulphs) PCA-Mix (C3G, PCA, and PCA conjugates), gallic acid glucuronides (GA-GlcAs), methylgallates (MethGA), and GA-Mix (D3G, gallic acid, and gallic acid conjugates). Treatments were conducted in triplicates and the experiments were repeated two times.

In this study, the direct effect of anthocyanin parent compounds and their metabolites on PON1 arylesterase activity was examined using two different phenotypes, PON-RR and PON-QQ (Figure 4A–D). The findings demonstrated that neither C3G or D3G affected the arylesterase activity at either concentration of the PON-RR or PON-QQ enzymes (Figure 4A). Likewise, none of the tested metabolites either the known, the potential or the phase-II conjugates affected the arylesterase activity, except for gallic acid, phloroglucinol, and cyanidin (Figure 4B–D).

Gallic acid at the higher concentration (10 µM) significantly increased PON-QQ arylesterase (but not PON-RR) by 9% compared to the control (Figure 4B, *p* ≤ 0.001). Similarly, phloroglucinol at both 1 and 10 µM increased PON-QQ (but not PON-RR) but by only 4% (Figure 4C, *p* ≤ 0.001). On the other hand, the high concentration of cyanidin (10 µM) but not the low concentration slightly decreased PON-RR by 6% (Figure 4C, *p* ≤ 0.001). Although statistically significant effects were observed for gallic acid, phloroglucinol and cyanidin, the effects were very modest.

#### 3.2.3. Effect of Anthocyanins and Their Metabolites on PON1 Lactonase Activity

**Figure 5:**
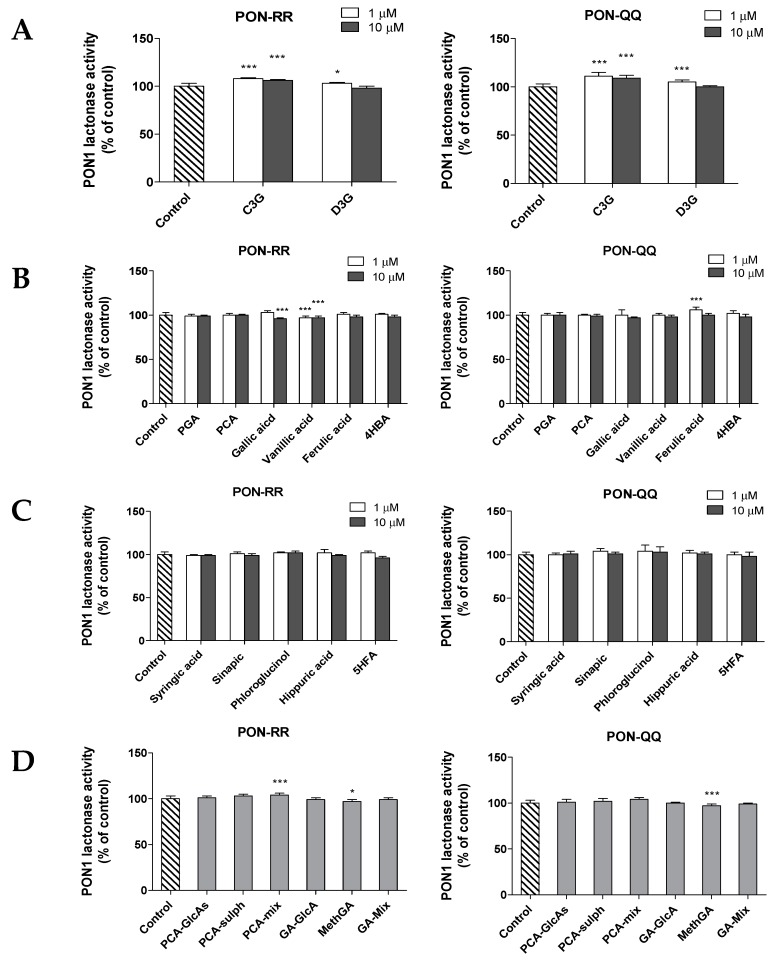
Effect of anthocyanins parent compounds (**A**), their known human metabolites (**B**), their potential/predicted metabolites (**C**), and phase-II conjugates (**D**) on lactonase activity of PON1 R192R (PON-RR) and Q192Q (PON-QQ) phenotypes. The % of change in activity calculated relative to the control (DMSO). Data are shown as means ± SD. * *p* ≤ 0.05 and *** *p* ≤ 0.001 as compared to control using one-way ANOVA coupled with Dunnett’s multiple comparison test. Treatments were: cyanidin-3-glucoside (C3G), delphinidin-3-glucoside (D3G), phloroglucinaldehyde (PGA), protocatechuic acid (PCA), 4-hydroxybenzaldehyde (4HBA), 5-hydroxyferulic acid (5HFA), PCA-glucuronides (PCA-GlcAs), PCA-sulphates (PCA-Sulphs) PCA-Mix (C3G, PCA and PCA conjugates), gallic acid glucuronides (GA-GlcAs), methylgallates (MethGA), and GA-Mix (D3G, gallic acid and gallic acid conjugates). Treatments were conducted in triplicates and the experiments were repeated two times.

As shown in Figure 5A–D, a few of the tested anthocyanins and their metabolites caused significant changes in PON1 lactonase activity, although the changes were not substantial. C3G had a very small but significant effect on the enzyme activity of both PON-RR and PON-QQ phenotypes. However, PON-QQ phenotype lactonase activity was affected more with increases of 11% and 9% at concentrations of 1 and 10 µM, respectively (*p* ≤ 0.001), while the lactonase activity of PON-RR increased by 6% and 8% at concentrations of 1 and 10 µM of C3G, respectively (*p* ≤ 0.001, Figure 5A). On the other hand, only a modest increase (5%) in PON-RR lactonase activity was observed with D3G (1 µM) (Figure 5A, *p* ≤ 0.001). Moreover, most of the tested anthocyanin metabolites did not cause significant changes, except for small changes in response to treatment with gallic acid, vanillic acid, and ferulic acid (Figure 5B, *p* ≤ 0.001).

**Figure 6:**
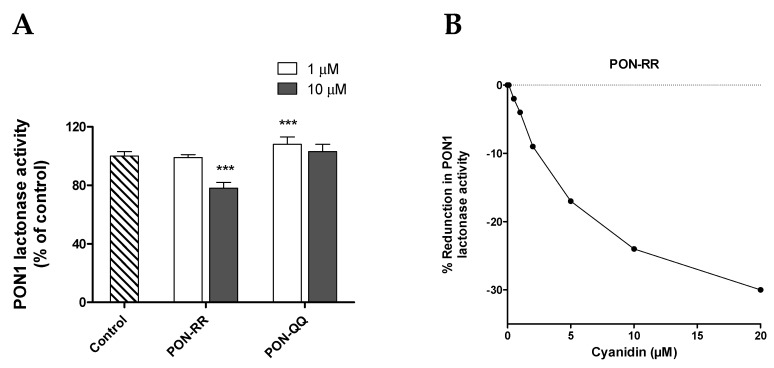
Effect of cyanidin on lactonase activity of PON1 R192R (PON-RR) and Q192Q (PON-QQ) phenotypes (**A**) and the dose response of PON-RR lactonase activity in the presence of cyanidin (**B**). The % of change in activity calculated relative to the control (DMSO). Data are shown as means ± SD. *** *p* ≤ 0.001 as compared to control using one-way ANOVA coupled with Dunnett’s multiple comparison test. % reduction in activity calculated relative to the control treatment. Treatments were conducted in triplicates and the experiments were repeated two times.

The only substantial effect was observed with cyanidin (Figure 6A,B). Unexpectedly, cyanidin at 10 µM significantly decreased PON-RR lactonase activity but not PON-QQ by 22% (*p* ≤ 0.001), while the lower concentration of cyanidin did not cause a change (Figure 6A). This finding was confirmed by doing a dose-response curve for cyanidin with PON-RR enzyme. As shown in Figure 6B, cyanidin decreased lactonase activity of PON-RR in a dose-dependent manner with about 30% reduction observed at the highest concentration (20 µM cyanidin). However, the lower more physiological concentrations of cyanidin (0.1, 0.5 µM, 1 µM) did not affect PON-RR activity. On the other hand, PON-QQ appeared to respond differently when treated with cyanidin: The low concentration of cyanidin (1 µM) but not the high one slightly increased PON-QQ by 8% (Figure 6A, *p* ≤ 0.001). However, it should be taken into account that the magnitude of effects where statistically significant effects were detected were modest (typically 4%−11%).

In summary, the data presented in this study do not support the notion that anthocyanins and/or their metabolites significantly affect *PON1* gene promoter activity or change the activity of PON1 enzymes. Even though some statistically significant changes were detected, the changes were very modest and probably lack clinical importance.

## 4. Discussion

The overall findings of the work reported here are that neither intact anthocyanins and anthocyanidins, nor their many phenolic degradation products, nor the tested phase‑II-conjugates that are the forms found in human blood caused any substantive activation of the PON1 promoter or in the arylesterase or lactonase activities of the major isoforms of PON1.

Previously reported data from epidemiological studies and from dietary interventions with human participants and animal models suggests that anthocyanins have the potential to decrease the risk of CVD [49,50,51]. In addition, there are numerous reports describing putative biological activities of anthocyanins based on treating, for example, cultured human cells with anthocyanins, typically at supraphysiological concentrations. The vast majority of these reported in vitro studies have examined only the activity of the intact form of anthocyanins and ignored the fact that anthocyanins undergo extensive metabolism [52,53]. The unequivocal identity of the metabolites of C3G was recently established from an elegant human feeding study that used penta-^13^C-labelled C3G that allowed the source of the metabolites (A- or B-ring of the anthocyanidin) to be established [37,38]. In addition, a number of metabolites of D3G and other trihydroxylated- and methylated-B ring anthocyanins have been reported, although anthocyanin-rich dietary sources were used instead of pure or isotope-labelled compounds [54]. The quantity of the parent (un-metabolized) ^13^C-C3G was very low (about 0.147 µm of ^13^C-labelled C3G), while the concentration of phenolic metabolites ranged from 0.1 to 2 µM with the cumulative concentration of metabolites reaching 10 µM [38,55] suggesting that the high bioactivity of anthocyanins is more likely to be mediated by the metabolites. However, the activities of most of these metabolites are unexplored or have only been reported at non- physiological doses, even up to 2.2 mM [56]. The present study, therefore, investigated for the first time the possible effects of known and potential metabolites of C3G and D3G including their phase-II-conjugates relative to their parent compounds at physiologically relevant concentrations, individually and in mixtures, on *PON1* gene expression and enzyme activities. In addition, the research presented in this paper is the first to the best of our knowledge to consider the potential interaction between PON1 genotype and the dietary anthocyanins using two different phenotypes corresponding to Q192Q and R192R genotype.

As the consumption of anthocyanins or anthocyanin-rich beverages has been reported to increase serum PON1 activity in some human studies [27,28], the underlying hypothesis of this research was that the increase in PON1 activity observed in these human studies was caused by the anthocyanins or their gut microbiota metabolites interacting with *PON1* gene expression or enzyme activity and increasing the enzyme concentration and/or the enzyme activity. Here, we screened the effects not just of the anthocyanins but also of most of the major metabolites of these compounds that are products of gut microbiota metabolism. We aimed to connect the compounds to the effect and identify which compounds caused the increase in activity and/or gene expression and at which concentrations. However, the data showed that none of these compounds at physiologically relevant concentrations (1–10 µM) caused significant changes in either PON1 promoter activity or arylesterase and lactonase activities, regardless of the phenotype, except for some small effects that likely lack clinical importance. The data presented here does not support the notion that there is a direct interaction between anthocyanins and/or their metabolites with the PON1 protein or the expression of the *PON1* gene. It is possible that the increase in PON1 activity in human interventions reported previously [27,28] was due to a secondary effect(s) of consuming anthocyanins. For example, the anthocyanins/metabolites might cause changes in the gut microbiome or interact with other organs such as the liver, but it is the anthocyanin-induced changes in the function of these organs that somehow caused the levels of PON1 activity to increase.

With regard effects on expression of the *PON1* gene, we have only reported effects on the PON1 promotor activation using a reporter assay. We attempted to assess the effects of the treatments on the abundance of PON1 transcripts in the classic human hepatic cell line HepG2 using qRT-PCR. However, we were not able to detect significant expression of the *PON1* gene in HepG2 cells in our laboratory. In keeping with this observation, we were also not able to detect PON1 activity in culture supernatants (secreted PON1, Figure S1) or cell homogenates (intracellular PON1, Figure S2). Details of the experiments with HepG2 cells are provided in the supplementary information (Supplementary information 1). Others have reported the use of a PON1-Huh7 transfected cell line and reported changes in PON1 promoter activity in response to treatment with several phenolic compounds such as curcumin, resveratrol, quercetin and isorhamnetin [30,33]. Gallic acid increased PON1 promoter activity by 8.5-fold, however this was in response to a very high and non-physiological concentration of gallic acid (360 µM) [57]. Here we report the effects of a physiological concentration (1 µM) and a slightly higher concentration (10 µM), but we observed no significant effects.

Very little is currently known of the effects of direct interaction between anthocyanins or their metabolites with the PON1 enzyme. The only available report to date is concerned with the effect of naringin, which caused a decrease in PON1 arylesterase activity after incubation with isolated PON1 enzyme [13]; therefore all the data presented here are novel. The only other reports we are aware of describe the effects of phenolic compounds such as resveratrol, quercetin, punicalagin and ellagic acid on PON-1 enzyme secretion using the Huh7 cell line [30,33]. We attempted to use the HepG2 cell line to study the effect of treatments on enzyme secretion, but we were not able to detect any secreted or cell-based PON1 activity in our laboratory (Supplementary information 1).

PON1 lactonase is the physiologically relevant activity that is associated with its biological functions [26,58,59]. Impairing the lactonase activity of PON1, e.g., via mutations of its catalytic dyad, diminished the ability of the enzyme to prevent LDL oxidation, reduced HDL-mediated cholesterol efflux, and abolished lysophosphatidylcholine (LPC) formation from phospholipid in macrophages, suggesting that the actual biological antiatherogenic functions of PON1 may be mediated by its lactonase activity [59]. However, paraoxonase and arylesterase were the only activities that had been reported previously from studies of polyphenols on PON1. To the best of our knowledge, the current study is the first to examine the effect of anthocyanins on PON1 lactonase activity and therefore provides new information regarding effects of anthocyanins on PON1 activity and by association with HDL function. Although the data presented here does not support the notion that direct interactions between anthocyanins and/or their metabolites affect the lactonase or arylesterase activity of PON1, it is not conclusive. The purified enzyme used here is not in its native environment which would include the presence of HDL and the ApoA1 protein, and this may affect the enzyme structural conformation and its stability and therefore its activity [60,61,62,63,64]. In theory it would be possible to use instead serum as a source of PON1. However, this may prove problematic because serum may contain other compounds such as lipoproteins that may bind with anthocyanins and conceal or inhibit their effects [65,66,67].

On the other hand, cyanidin decreased PON1 lactonase activity, but at super physiological concentrations and not at lower more physiological concentrations. However, the negative effect of cyanidin may be physiologically unimportant. In fact, cyanidin was not detected in serum in humans after consumption of ^13^C-labelled C3G. Only traces of cyanidin conjugates were detected in urine in the same study suggesting that the existence of cyanidin in serum in concentrations higher than 0.1 μM is not achievable [38]. Furthermore, cyanidin is extremely unstable at high pH, with about 50% instantaneous loss of the parent structure at pH 7.4, which was lower than the pH of the assay buffer (pH = 8) used in the current study. This suggests that the reduction in PON1 lactonase activity was not mediated directly by cyanidin but may though one or more of its intermediates [68].

As far as the authors are aware, this is the first study to report the interaction between anthocyanins and their metabolites, PON1 genotype and PON1 activities. None of the previous reports that investigated the effects of anthocyanins and other polyphenols on PON1 activities have considered the association between PON1 polymorphisms and its activity [27,28]. Here we used two enzyme isoenzymes that correspond with two functional Q192R polymorphisms, PON-QQ and PON-RR. At high concentrations, cyanidin decreased PON1 lactonase activity of the PON-RR isoenzyme, but it increased the lactonase activity of PON-QQ. These observations support the notion that individuals respond differently to treatment according to their PON1 genotype, and therefore the genetic variance should be taken into account. Although both phenotypes responded similarly to the other anthocyanins/metabolites, this does not preclude that these metabolites may interact and affect the enzyme activities with other genotypes other than the Q192R genotype. In a previous nutrigenomic study, a significant association between anthocyanin intake and increased HDL levels was reported in participants with some PON1 genotypes but not with others [69]. The study investigated interactions between SNPs in the *PON1* gene and polyphenols intake on HDL and other biomarkers of CVD risk. Of 18 independent tagging SNPs that were tested, four SNPs (rs854549, rs854552, rs854571, and rs854572) showed a significant association with increased circulating HDL levels in people consuming higher quantities of polyphenols and anthocyanins [69]. At present there is no commercial source of purified isoenzymes corresponding to these genotypes to understand how these genotypes respond to anthocyanin treatments. However, undertaking a human intervention with large population and categorizing the participants into different group based on their genotype and studying the enzyme activities after anthocyanin intervention would provide a better insight into the interaction between anthocyanins, PON1 activities and PON1 genotype.

Although the data presented in this report are novel, there are several limitations. Measuring the effects of the polyphenols on promoter activity rather than gene expression (transcript levels) is one weakness. The reporter assay may not faithfully recapitulate the native chromosome environment [70]. The treatments may affect the stability of mRNA or other posttranscriptional regulators of the *PON1* gene without affecting the promotor activity, which will not be detected using the reporter assay. In addition, the effects of the anthocyanins and their metabolites on enzyme secretion has not been investigated in the current study due the authors not having a suitable model. It is possible that the treatments do not change *PON1* gene expression or the activity of the enzyme, but instead affect cell permeability or the cellular transporters that would lead to an increase in PON1 enzyme levels in serum. In our hands, HepG2 cells did not secrete detectable quantities of the enzyme, excluding its use as a suitable model for this purpose. Should a fit-for-purpose cultured cell model be available, future work should focus on the effect of anthocyanins and their metabolites on PON1 enzyme secretion to ensure that this potential mechanism of actions is also investigated.

In conclusion, the novel data presented in this study do not support the notion that anthocyanins and/or their metabolites significantly affect *PON1* gene promoter activity or change the activities of PON1 isoenzymes. Even though some statistically significant changes were detected, the changes were very modest and probably lack clinical importance. However, it is feasible that these compounds alternatively affect PON1 secretion or interact with different PON1 genotypes rather than those were reported here, and this should be considered in future studies.

## Supplementary Materials

The following are available online at https://www.mdpi.com/2072-6643/11/12/2872/s1. Supplementary information 1: PON1 gene expression and enzyme activity in HepG2 cell. Figure S1: Secreted PON1 arylesterase activity using HepG2 cell, Figure S2: Cell-based PON1 arylesterase activity using HepG2 cell.

## References

[1] Hansen A.S., Marckmann P., Dragsted L.O., Finné Nielsen I.L., Nielsen S.E., Gronbæk M. (2005). Effect of red wine and red grape extract on blood lipids, haemostatic factors, and other risk factors for cardiovascular disease. Eur. J. Clin. Nutr..

[2] Gurrola-Díaz C.M., García-López P.M., Sánchez-Enríquez S., Troyo-Sanromán R., Andrade-González I., Gómez-Leyva J.F. (2010). Effects of Hibiscus sabdariffa extract powder and preventive treatment (diet) on the lipid profiles of patients with metabolic syndrome (MeSy). Phytomedicine.

[3] Qin Y., Xia M., Ma J., Hao Y., Liu J., Mou H., Cao L., Ling W. (2009). Anthocyanin supplementation improves serum LDL- and HDL-cholesterol concentrations associated with the inhibition of cholesteryl ester transfer protein in dyslipidemic subjects. Am. J. Clin. Nutr..

[4] Rodriguez-Mateos A., Heiss C., Borges G., Crozier A. (2014). Berry (Poly)phenols and Cardiovascular Health. J. Agric. Food Chem..

[5] Wilson P.W., Abbott R.D., Castelli W.P. (1988). High density lipoprotein cholesterol and mortality. The Framingham Heart Study. Arterioscler. An. Off. J. Am. Hear. Assoc. Inc..

[6] Shah P.K., Amin J. (1992). Low high density lipoprotein level is associated with increased restenosis rate after coronary angioplasty. Circulation.

[7] Gordon D.J., Probstfield J.L., Garrison R.J., Neaton J.D., Castelli W.P., Knoke J.D., Jacobs D.R., Bangdiwala S., Tyroler H.A. (1989). High-density lipoprotein cholesterol and cardiovascular disease. Four prospective American studies. Circulation.

[8] Haas M.J., Mooradian A.D. (2010). Regulation of high-density lipoprotein by inflammatory cytokines: Establishing links between immune dysfunction and cardiovascular disease. Diabetes. Metab. Res. Rev..

[9] Raffield L.M., Cox A.J., Hsu F.C., Ng M.C.Y., Langefeld C.D., Carr J.J., Freedman B.I., Bowden D.W. (2013). Impact of HDL genetic risk scores on coronary artery calcified plaque and mortality in individuals with type 2 diabetes from the Diabetes Heart Study. Cardiovasc. Diabetol..

[10] Shah P.K. (2013). Jekyll and Hyde of HDL: A lipoprotein with a split personality. Eur. Heart, J..

[11] Ikhlef S., Berrougui H., Simo O.K., Zerif E., Khalil A. (2017). Human paraoxonase 1 overexpression in mice stimulates HDL cholesterol efflux and reverse cholesterol transport. PLOS ONE.

[12] Rosenson R.S., Brewer H.B., Davidson W.S., Fayad Z.A., Fuster V., Goldstein J., Hellerstein M., Jiang X.C., Phillips M.C., Rader D.J. (2012). Cholesterol efflux and atheroprotection: Advancing the concept of reverse cholesterol transport. Circulation.

[13] Mahooz A., Rashidi M.-R., Nouri M. (2011). Naringenin is an inhibitor of human serum paraoxonase (PON1): An in vitro study. J. Clin. Lab. Anal..

[14] Gugliucci A., Menini T. (2015). Paraoxonase 1 and HDL maturation. Clin. Chim. Acta.

[15] Sumegová K., Nagyová Z., Waczulíková I., Žitňanová I., Ďuračková Z., Sumegova K., Nagyova Z., Waczulikova I., Zitnanova I., Durackova Z.L.B.-S. (2007). Activity of Paraoxonase 1 and Lipid Profile in Healthy Children. Physiol. Res..

[16] Turgut Cosan D., Colak E., Saydam F., Yazıcı H.U., Degirmenci I., Birdane A., Colak E., Gunes H.V. (2016). Association of paraoxonase 1 (PON1) gene polymorphisms and concentration with essential hypertension. Clin. Exp. Hypertens..

[17] ME L., YC L., RT L., YS W., Hsi E., HF L., KC C., SH J., Liu M.-E., Liao Y.-C. (2013). A functional polymorphism of PON1 interferes with microRNA binding to increase the risk of ischemic stroke and carotid atherosclerosis. Atherosclerosis.

[18] Brophy V.H., Jampsa R.L., Clendenning J.B., McKinstry L.A., Jarvik G.P. (2001). Effects of 5′ regulatory-region polymorphisms on paraoxonase-gene (PON1) expression. Am. J. Hum. Genet..

[19] Campo S., Sardo M.A., Trimarchi G., Bonaiuto M., Fontana L., Castaldo M., Bonaiuto A., Saitta C., Bitto A., Manduca B. (2004). Association between serum paraoxonase (PON1) gene promoter T(-107)C polymorphism, PON1 activity and HDL levels in healthy Sicilian octogenarians. Exp. Gerontol..

[20] Santos F.G., Becker M.K., Corrêa V.S., Garcia D.N., Vale S.C., Crespo-Ribeiro J.A., Barros C.C., Schneider A. (2016). The effect of the paraoxonase 1 (PON1) T(-107)C polymorphism on serum PON1 activity in women is dependent on fatty acid intake. Nutr. Res..

[21] Gupta N., Singh S., Maturu V.N., Sharma Y.P., Gill K.D. (2011). Paraoxonase 1 (PON1) polymorphisms, haplotypes and activity in predicting CAD risk in North-West Indian Punjabis. PLoS ONE.

[22] Aviram M., Hardak E., Vaya J., Mahmood S., Milo S., Hoffman A., Billicke S., Draganov D., Rosenblat M. (2000). Human Serum Paraoxonases (PON1) Q and R Selectively Decrease Lipid Peroxides in Human Coronary and Carotid Atherosclerotic Lesions. Circulation.

[23] Mackness B., Mackness M.I., Arrol S., Turkie W., Julier K., Abuasha B., Miller J.E., Boulton A.J., Durrington P.N. (1998). Serum paraoxonase (PON1) 55 and 192 polymorphism and paraoxonase activity and concentration in non-insulin dependent diabetes mellitus. Atherosclerosis.

[24] Quillen E.E., Rainwater D.L., Dyer T.D., Carless M.A., Curran J.E., Johnson M.P., Göring H.H.H., Cole S.A., Rutherford S., MacCluer J.W. (2012). Novel Associations of Nonstructural Loci with Paraoxonase Activity. J. Lipids.

[25] Brophy V.H., Hastings M.D., Clendenning J.B., Richter R.J., Jarvik G.P., Furlong C.E. (2001). Polymorphisms in the human paraoxonase (PON1) promoter. Pharmacogenetics.

[26] Ceron J.J., Tecles F., Tvarijonaviciute A. (2014). Serum paraoxonase 1 (PON1) measurement: An update. BMC Vet. Res..

[27] Zhu Y., Huang X., Zhang Y., Wang Y., Liu Y., Sun R., Xia M. (2014). Anthocyanin supplementation improves HDL-Associated paraoxonase 1 activity and enhances cholesterol efflux capacity in subjects with hypercholesterolemia. J. Clin. Endocrinol. Metab..

[28] Aviram M., Rosenblat M., Gaitini D., Nitecki S., Hoffman A., Dornfeld L., Volkova N., Presser D., Attias J., Liker H. (2004). Pomegranate juice consumption for 3 years by patients with carotid artery stenosis reduces common carotid intima-media thickness, blood pressure and LDL oxidation. Clin. Nutr..

[29] Esatbeyoglu T., Rodríguez-Werner M., Schlösser A., Winterhalter P., Rimbach G. (2017). Fractionation, enzyme inhibitory and cellular antioxidant activity of bioactives from purple sweet potato (Ipomoea batatas). Food Chem..

[30] Boesch-Saadatmandi C., Egert S., Schader C., Coumoul X., Barouki R., Muller M.J., Wolffram S., Rimbach G. (2010). Effect of quercetin on paraoxonase 1 activity - studies in cultured cells, mice and humans. J. Physiol. Pharmacol..

[31] Fuhrman B., Aviram M. (2002). Preservation of paraoxonase activity by wine flavonoids: Possible role in protection of LDL from lipid peroxidation. Ann. N. Y. Acad. Sci..

[32] Ustundag B., Bahcecioglu I.H., Sahin K., Duzgun S., Koca S., Gulcu F., Ozercan I.H. (2007). Protective effect of soy isoflavones and activity levels of plasma paraoxonase and arylesterase in the experimental nonalcoholic steatohepatitis model. Dig. Dis. Sci..

[33] Gouedard C., Barouki R., Morel Y., Gouédard C., Barouki R., Morel Y. (2004). Induction of the paraoxonase-1 gene expression by resveratrol. Arter. Thomb. Vasc. Biol..

[34] Gupta N., Kandimalla R., Priyanka K., Singh G., Gill K.D., Singh S. (2014). Effect of resveratrol and nicotine on PON1 gene expression: In vitro study. Indian, J. Clin. Biochem..

[35] Gouedard C., Barouki R., Morel Y., Gouédard C., Barouki R., Morel Y., Gouedard C., Barouki R., Morel Y. (2004). Dietary polyphenols increase paraoxonase 1 gene expression by an aryl hydrocarbon receptor-dependent mechanism. Mol. Cell Biol..

[36] Krga I., Monfoulet L.E., Konic-Ristic A., Mercier S., Glibetic M., Morand C., Milenkovic D. (2016). Anthocyanins and their gut metabolites reduce the adhesion of monocyte to TNFalpha-activated endothelial cells at physiologically relevant concentrations. Arch. Biochem Biophys.

[37] Czank C., Cassidy A., Zhang Q., Morrison D.J., Preston T., Kroon P.A., Botting N.P., Kay C.D. (2013). Human metabolism and elimination of the anthocyanin, cyanidin-3-glucoside: A 13C-tracer study. Am. J. Clin. Nutr..

[38] De Ferrars R.M., Czank C., Zhang Q., Botting N.P., Kroon P.A., Cassidy A., Kay C.D. (2014). The pharmacokinetics of anthocyanins and their metabolites in humans. Br. J. Pharmacol..

[39] Schader C., Schiborr C., Frank J., Rimbach G. (2011). Curcumin induces paraoxonase 1 in cultured hepatocytes in vitro but not in mouse liver in vivo. Br. J. Nutr..

[40] Van Rymenant E., Grootaert C., Beerens K., Needs P.W., Kroon P.A., Kerimi A., Williamson G., García-Villalba R., González-Sarrías A., Tomas-Barberan F. (2017). Vasorelaxant activity of twenty-one physiologically relevant (poly)phenolic metabolites on isolated mouse arteries. Food Funct..

[41] Jaichander P., Selvarajan K., Garelnabi M., Parthasarathy S. (2008). Induction of paraoxonase 1 and apolipoprotein A-I gene expression by aspirin. J. Lipid Res..

[42] Tvarijonaviciute A., Tecles F., Caldin M., Tasca S., Cerón J. (2012). Validation of spectrophotometric assays for serum paraoxonase type-1 measurement in dogs. Am. J. Vet. Res..

[43] Browne R.W., Koury S.T., Marion S., Wilding G., Muti P., Trevisan M. (2007). Accuracy and biological variation of human serum paraoxonase 1 activity and polymorphism (Q192R) by kinetic enzyme assay. Clin. Chem..

[44] Martinelli N., Girelli D., Olivieri O., Guarini P., Bassi A., Trabetti E., Friso S., Pizzolo F., Bozzini C., Tenuti I. (2009). Novel serum paraoxonase activity assays are associated with coronary artery disease. Clin. Chem. Lab. Med..

[45] Gaidukov L., Tawfik D.S. (2007). The development of human sera tests for HDL-bound serum PON1 and its lipolactonase activity. J. Lipid Res..

[46] Ferre N., Feliu A., Garcia-Heredia A., Marsillach J., Paris N., Ferré N., Feliu A., García-Heredia A., Marsillach J., París N. (2013). Impaired paraoxonase-1 status in obese children. Relationships with insulin resistance and metabolic syndrome. Clin. Biochem..

[47] Khersonsky O., Tawfik D.S. (2006). Chomogenic and fluorogenic assays for the lactonase activity of serum paraoxonases. Chem. Bio. Chem..

[48] Esatbeyoglu T., Ulbrich K., Rehberg C., Rohn S., Rimbach G. (2015). Thermal stability, antioxidant, and anti-inflammatory activity of curcumin and its degradation product 4-vinyl guaiacol. Food Funct..

[49] Wallace T.C., Slavin M., Frankenfeld C.L. (2016). Systematic review of anthocyanins and markers of cardiovascular dise. Nutrients.

[50] Cassidy A., Bertoia M., Chiuve S., Flint A., Forman J., Rimm E.B. (2016). Habitual intake of anthocyanins and flavanones and risk of cardiovascular disease in men. Am. J. Clin. Nutr..

[51] Blesso C.N. (2019). Dietary Anthocyanins and Human Health. Nutrients.

[52] Kolehmainen M., Mykkänen O., Kirjavainen P.V., Leppänen T., Moilanen E., Adriaens M., Laaksonen D.E., Hallikainen M., Puupponen-Pimiä R., Pulkkinen L. (2012). Bilberries reduce low-grade inflammation in individuals with features of metabolic syndrome. Mol. Nutr. Food Res..

[53] Karlsen A., Paur I., Bohn S.K., Sakhi A.K., Borge G.I., Serafini M., Erlund I., Laake P., Tonstad S., Blomhoff R. (2010). Bilberry juice modulates plasma concentration of NF-κB related inflammatory markers in subjects at increased risk of CVD. Eur. J. Nutr..

[54] Mueller D., Jung K., Winter M., Rogoll D., Melcher R., Richling E. (2017). Human intervention study to investigate the intestinal accessibility and bioavailability of anthocyanins from bilberries. Food Chem..

[55] Amin H.P., Czank C., Raheem S., Zhang Q., Botting N.P., Cassidy A., Kay C.D. (2015). Anthocyanins and their physiologically relevant metabolites alter the expression of IL-6 and VCAM-1 in CD40L and oxidized LDL challenged vascular endothelial cells. Mol. Nutr. Food Res..

[56] Youdim K.A., Martin A., Joseph J.A. (2000). Incorporation of the elderberry anthocyanins by endothelial cells increases protection against oxidative stress. Free Radic. Biol. Med..

[57] Khateeb J., Gantman A., Kreitenberg A.J., Aviram M., Fuhrman B. (2010). Paraoxonase 1 (PON1) expression in hepatocytes is upregulated by pomegranate polyphenols: A role for PPAR-gamma pathway. Atherosclerosis.

[58] Rupérez A.I., López-Guarnido O., Gil F., Olza J., Gil-Campos M., Leis R., Tojo R., Cañete R., Gil A., Aguilera C.M. (2013). Paraoxonase 1 activities and genetic variation in childhood obesity. Br. J. Nutr..

[59] Rosenblat M., Gaidukov L., Khersonsky O., Vaya J., Oren R., Tawfik D.S., Aviram M. (2006). The catalytic histidine dyad of high density lipoprotein-associated serum paraoxonase-1 (PON1) is essential for PON1-mediated inhibition of low density lipoprotein oxidation and stimulation of macrophage cholesterol efflux. J. Biol. Chem..

[60] Levy D., Reichert C.O., Bydlowski S.P., Levy D., Reichert C.O., Bydlowski S.P. (2019). Paraoxonases activities and polymorphisms in elderly and old-age diseases: An overview. Antioxidants.

[61] Phuntuwate W., Suthisisang C., Koanantakul B., Mackness M.I., Mackness B. (2005). Paraoxonase 1 status in the Thai population. J. Hum. Genet..

[62] Atrahimovich D., Vaya J., Tavori H., Khatib S. (2012). Glabridin protects paraoxonase 1 from linoleic acid hydroperoxide inhibition via specific interaction: A fluorescence-quenching study. J. Agric. Food Chem..

[63] Deakin S., Leviev I., Gomaraschi M., Calabresi L., Franceschini G., James R.W. (2002). Enzymatically active paraoxonase-1 is located at the external membrane of producing cells and released by a high affinity, saturable, desorption mechanism. J. Biol. Chem..

[64] Graves T.L., Scott J.E. (2008). A High Thoughput Serum Paraoxonase Assay for Discovery of Small Molecule Modulators of PON1 Activity. Curr. Chem. Genomics.

[65] Latruffe N., Menzel M., Delmas D., Buchet R., Lançon A. (2014). Compared Binding Properties between Resveratrol and Other Polyphenols to Plasmatic Albumin: Consequences for the Health Protecting Effect of Dietary Plant Microcomponents. Molecules.

[66] Nozaki A., Hori M., Kimura T., Ito H., Hatano T. (2009). Interaction of Polyphenols with Proteins: Binding of (−)-Epigallocatechin Gallate to Serum Albumin, Estimated by Induced Circular Dichoism. Chem. Pharm. Bull. (Tokyo)..

[67] Cao H., Liu X., Ulrih N.P., Sengupta P.K., Xiao J. (2019). Plasma protein binding of dietary polyphenols to human serum albumin: A high performance affinity chomatography approach. Food Chem..

[68] Kay C.D., Kroon P.A., Cassidy A. (2009). The bioactivity of dietary anthocyanins is likely to be mediated by their degradation products. Mol. Nutr. Food Res..

[69] Rizzi F., Conti C., Dogliotti E., Terranegra A., Salvi E., Braga D., Ricca F., Lupoli S., Mingione A., Pivari F. (2016). Interaction between polyphenols intake and PON1 gene variants on markers of cardiovascular disease: A nutrigenetic observational study. J. Transl Med..

[70] Atkinson T.J., Halfon M.S. (2014). Regulation of gene expression in the genomic context. Comput. Struct. Biotechnol. J..

