# Supplementary material for: The Effects of Anthocyanins and Their Microbial Metabolites on the Expression and Enzyme Activities of Paraoxonase 1, an Important Marker of HDL Function
Source: Nutrients. 2019 Nov 24;11(12):2872. doi: 10.3390/nu11122872 (PMC6950676; doi:10.3390/nu11122872)
Supplement: Supplementary file 1 [file nutrients-11-02872-s001.pdf]

**PON1 gene expression and enzyme activity in HepG2 cell.**

## 1. Material and methods

*Cell culture*

Human hepatoma cell line HepG2 was maintained in minimum essential medium with Earle's Balanced Salts (EMEM) containing 2mM L-glutamine supplemented with 10% FBS, penicillin (100 U/ml) and streptomycin (100 µg/ml) in a 75T flask at 37°C in a humidified incubator under atmosphere containing 5% $CO_2$.

### *1.1. Secreted PON1 activity*

HepG2 were seeded in 6-well plates (0.5 x $10^6$ cell/well) using complete media and incubated for 48 hr. The medium was removed, and the cells were washed twice with pre-warmed PBS. The cell layer was then treated with the compound of interest using serum-free medium for 48 h. The media, was collected and used as source of PON1 enzyme. PON1 arylesterase activity was measured as described early in the material and methods section in the main article.

### *1.2. Cell-based PON1 activity*

The cells were seeded as previously described. After 48 h, the cells were incubated with the compound of interest prepared in media for an additional 48 h. At the end of the incubation period, the medium was discarded, and the cells were washed twice with pre-warmed PBS. Cell-based PON1 activity was measured by adding the reaction mixture that contained the substrate to the cell layer and monitoring the change in absorbance. The reaction was initiated by the addition of 1 ml of buffer containing p-nitrophenyl acetate to yield a final concentration of 1mM. The reaction mixture was incubated at 37°C and the absorbance was recorded at 410 nm at the end of a 20 min incubation. 1 ml of reaction mixture containing 100 µM 2-hydroxyquinoline (2-HQ) was added, under the same condition to a number of treated wells to correct for PON-1 specific activity. Autohydrolysis background activity was also determined by the addition of reaction mixture to cell-free wells.

### *1.3. PON1 gene expression in HepG2*

The cells were treated with DMSO and 10 µM of PCA as mentioned earlier. RNA extraction was performed using RNeasy® Mini Kit (Qiagen Ltd, Cat# 74106) according to the manufacturer's protocol. PON1 gene expression was performed as described elsewhere [1]. PON1 gene expression was normalized against GAPDH. The sequences of forward and reverse primers for PON1 were: 5`-TTCA CCCGATGGCAAGTATG-3` and 5`-ACGAGGGTATTAAAGTCAAGGG-3`, respectively.

## 2. Results

Toward the goal of developing an assay to test the effect of anthocyanins and their metabolites on PON1 gene expression, activity and secretion, the ability of HepG2 to express and secrete PON1 was tested.

### *2.1. Secreted PON1 activity.*

To investigate whether HepG2 secret PON1 to the media, HepG2 cells were treated with 10 µM PCA and DMSO for 48 h before collecting the media and use it as source of the enzyme. The absorbance obtained from treatment was similar to the absorbance from background (Blank 0.31 ± 0.002, DMSO 0.30 ± 0.006, PCA 0.30 ± 0.004) which means that HepG2 did not secreted any PON1 and the colour was attributed to the autohydrolysis. In an attempt to increase the secreted enzyme concentration, the cell number was increased (two times) and the volume of the media was also increased in the reaction mixture (9 times). In this experiment, 100 µM 2-HQ was used to calculate PON-1-derived activity. As shown in Figure S1, the secreted arylesterase activity increased, although the activity was not derived from PON1 as the activity in the presence and the absence of PON1 inhibitor was identical which indicates that HepG2 did not secret PON1. Probably, HepG2 synthesised the enzyme endogenously

but couldn't secret it as FBS-free media was used in the treatment so there were not any sources of HDL for the enzyme to be structured on. To test the possibility of the endogenous production of PON1, cell-based PON1 activity was measured.

### *2.2. Cell-based PON1*

In order to investigate whether HepG2 PON1 synthetizes endogenously, cell-based PON1 was measured. HepG2 cells were treated with DMSO and two concentration of aspirin (0.25 and 0.5 mM) which previously reported to induce cell-associated PON1 by 3-fold [2]. The assay buffer that contain the substrate was added directly to the cell layer in the presence and the absence of 2-HQ. As shown in Figure S2, it was obvious that there were more endogenous arylesterases than the secreted. The absorbance of cell-based arylesterases was about 1.8 unit, while, the secreted arylesterases was less than 0.9 unit (Figure S1 &S2). However, the data also showed that the cell-based arylesterases activity was not mediated by PON1 as the absorbance in presence and the absence of PON1 inhibitor was similar (Figure S2). In addition, none of the aspirin treatments increased PON1 production (Figure S2) suggesting that HepG2 cells model was not suitable for investigating the effects of treatments on PON1 enzyme secretion.

### *2.3. PON1 gene expression in HepG2*

Since, no secreted or cell-based PON1 activity was detected, the expression of PON1 gene was quantified to explore whether PON1 was expressed in HepG2 cell or not. mRNA transcription level of PON1 was very low with Ct values higher than 30 indicating that PON1 gene is lowly expressed in HepG2.

Overall, the HepG2 cell did not synthesize PON1 enzyme and the gene expression was very low suggesting that the used HepG2 cell was not suitable for testing the effects of anthocyanins and/or their metabolites on PON1 activities and gene expression. Instead, stably transfected cell line with PON1 promoter was used as a model and the promoter activity was measured using the reporter gene assay.

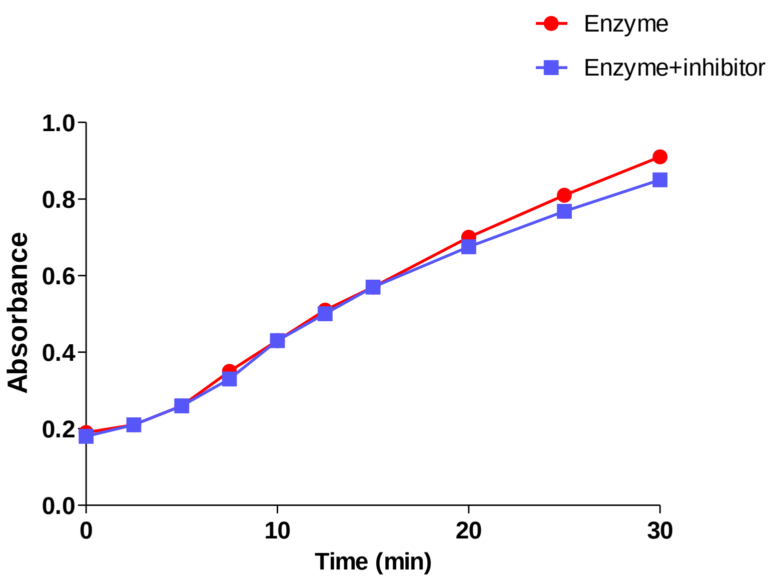


**Figure S1:** Secreted PON1 arylesterase activity using HepG2 cell. The arylesterase activity was measured using *p*-nitrophenyl acetate (1 mm) as a substrate with the presence or the absence of 100 μM of 2-HQ and monitoring the change in the absorbance at 410 nm. The measurement was corrected for background which was media collected from cell-free well.

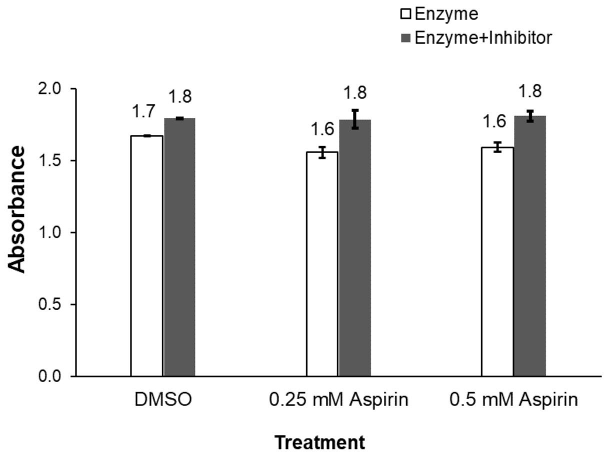


**Figure S2:** Cell-based PON1 arylesterase activity using HepG2 cell. Aspirin (0.25 and 0.5 mM) which previously reported to induce cell-associated PON1. After treatments, the cell layer was incubated for 20 min with 1 ml of reaction mixture that contain 1 mm p-nitrophenyl acetate with the presence and the absence of PON1 inhibitor (100 µM of 2-HQ). The measurement was corrected for background which was measurement for cell-free well. Data are shown as means ± SD. No significant differences were detected compared with control using one-way ANOVA coupled with Dunnett's multiple comparison test.
